# Household Transmission of SARS-CoV-2 from Humans to Pets, Washington and Idaho, USA

**DOI:** 10.3201/eid2812.220215

**Published:** 2022-12

**Authors:** Julianne Meisner, Timothy V. Baszler, Kathryn E. Kuehl, Vickie Ramirez, Anna Baines, Lauren A. Frisbie, Eric T. Lofgren, David M. de Avila, Rebecca M. Wolking, Dan S. Bradway, Hannah R. Wilson, Beth Lipton, Vance Kawakami, Peter M. Rabinowitz

**Affiliations:** University of Washington, Seattle, Washington, USA (J. Meisner, V. Ramirez, A. Baines, P.M. Rabinowitz);; Washington State University, Pullman, Washington, USA (T.V. Baszler, K.E. Kuehl, E.T. Lofgren, D.M. de Avila, R.M. Wolking, D.S. Bradway, H.R. Wilson);; Washington State Department of Health, Shoreline, Washington, USA (L.A. Frisbie);; Public Health Seattle and King County, Seattle (B. Lipton, V. Kawakami)

**Keywords:** severe acute respiratory syndrome coronavirus 2, SARS-CoV-2, coronaviruses, viruses, coronavirus disease, COVID-19, respiratory infections, human, pets, One Health, anthropozoonoses, household transmission, burden, risk factors, zoonoses, Washington, Idaho, United States

## Abstract

SARS-CoV-2 likely emerged from an animal reservoir. However, the frequency of and risk factors for interspecies transmission remain unclear. We conducted a community-based study in Idaho, USA, of pets in households that had >1 confirmed SARS-CoV-2 infections in humans. Among 119 dogs and 57 cats, clinical signs consistent with SARS-CoV-2 were reported for 20 dogs (21%) and 19 cats (39%). Of 81 dogs and 32 cats sampled, 40% of dogs and 43% of cats were seropositive, and 5% of dogs and 8% of cats were PCR positive. This discordance might be caused by delays in sampling. Respondents commonly reported close human‒animal contact and willingness to take measures to prevent transmission to their pets. Reported preventive measures showed a slightly protective but nonsignificant trend for both illness and seropositivity in pets. Sharing of beds and bowls had slight harmful effects, reaching statistical significance for sharing bowls and seropositivity.

Coronaviruses infect multiple mammal species, and SARS-CoV-2, the etiologic agent of COVID-19, likely jumped to humans from a mammal source (*1*). Although the virus is currently spreading person-to-person, the angiotensin converting enzyme-2 receptor involved in SARS-CoV-2 transmission is present in multiple species, and there are numerous reports of infections in pets (*2**–**4*). As of October 17, 2022, a total of 110 domestic cats and 95 domestic dogs in the United States had been reported by the US Department of Agriculture Animal and Plant Health Inspection Service to have SARS-CoV-2 infection (*5*).

Workplace transmission of SARS-CoV-2 between humans and animals has also been documented, including in zoos (felids and nonhuman primates), on mink farms (*6*,*7*), and at pet warehouses/pet shops (*8*,*9*). These findings are consistent with previous reports of SARS-CoV-1 infecting cats and ferrets, and laboratory studies demonstrating experimental SARS-CoV-2 infection of nonhuman primates, ferrets, hamsters, and rabbits (*10*). However, less is known about the frequency of and risk factors for SARS-CoV-2 transmission between humans and companion animals in a household setting. Furthermore, the natural history of SARS-CoV-2 infection in pets is poorly understood.

Given the close contact many persons have with their pets and the intimate nature of their shared environment, exacerbated during periods of quarantine or isolation, it is useful to clarify the role of companion animals in community infection patterns, including contribution to virus evolution and emergence of novel strains. In light of evidence from mink farms that animal-origin variants might contain spike gene mutations and other changes that could affect clinical features of infection (*11*,*12*), evidence suggesting mouse origins of the Omicron SARS-CoV-2 variant (*13*), and the recent decision in Hong Kong to cull 2,000 hamsters after a pet shop worker was infected (*14*), ongoing monitoring of SARS-CoV-2 transmission between humans and animals remains critical.

We report findings from the COVID-19 and Pets Study, a cross-sectional community-based study of pets in households of persons that had documented COVID-19 infection in Washington and Idaho, USA. The goal of the study was to describe the frequency of transmission between humans and animals within a household, and to determine human, animal, and environmental risk factors for that transmission, in a One Health framework.

## Methods

### Study Population and Design

The COHERE (*15*) and STROBE (*16*) statements were used to guide reporting of the findings and the preparation of this article. We defined a household as >1 persons >18 years of age living with >1 pet that does not live solely outdoors. Pets were defined as dogs, cats, ferrets, and hamsters, based on previous research documenting experimental COVID-19 infection in these species (*17*,*18*). We conducted this study in King, Snohomish, Yakima, Whitman, Pierce, Spokane, and Benton Counties in Washington and Latah County in Idaho during April 2020‒September 2021. The COVID-19 and Pets Study is a cross-sectional study with individual-level and household-level data collection. Study participation involved 2 components: an online survey, followed by animal sampling.

### Recruitment and Eligibility

Households were recruited through partnerships with other COVID-19 clinical trials and community studies, as well as by social media, word of mouth, community partners, and contact tracers from Public Health Seattle and King County during case investigation/contact tracing calls. We screened persons for eligibility by using the UW Research Electronic Data Capture (REDCap) system (*19*), a Health Insurance Portability and Accountability Act‒compliant web tool for clinical research, which had criteria including county of residence, pet ownership, and >1 household member with confirmed SARS-CoV-2 infection by PCR or antigen testing by a provider or laboratory. Animals with known fearful or aggressive behavior were excluded. However, other animals in the corresponding household were eligible.

### Ethics Statement

This study received ethics approval from the University of Washington Institutional Review Board (STUDY00010585) and the Office of Animal Welfare (PROTO201600308: 4355–01). We obtained informed consent by using REDCap or over the telephone with the study coordinator, after the nature and possible consequences of study involvement had been explained.

### Survey

A household member completed a survey before the sampling visit was scheduled. Human survey items included COVID-19 symptoms, onset, and severity; concurrent conditions; vaccination status, dates, and type; and reported COVID-19‒like illness of any other household members, including those without confirmatory testing. Animal survey items included veterinary clinical variables, history of illness compatible with SARS-CoV-2 infection, and contact with specific members of the household. Environmental survey items included type and size of home, type of flooring (e.g., carpet, wood), and availability of outdoor space for pets to roam.

At the sampling visit, the field team inquired about updates for human and animal household members, including new hospitalizations, symptoms, or COVID-19 diagnoses. The study team also reviewed SARS-CoV-2 test results to confirm date and positive result; self-test results were not accepted.

### Animal Sampling

A team of 2 study personnel, including at least 1 veterinarian, performed sampling in the home of a participant or at a veterinary hospital. No chemical restraint was used because of biosafety concerns, and no muzzles were used.

The team used species-appropriate restraint standard techniques for venipuncture and collection of 3 mL of blood into a labeled serum separator tube. Swab specimen samples collected from rostral nares and the caudal oropharynx were placed into 1 Primestore Molecular Transport Medium Tube (Longhorn Vaccines and Diagnostics LLC, https://lhnvd.com). A fecal swab specimen collected from the rectum was placed into a separate tube. All participants received educational information from the field team about measures to mitigate household COVID-19 transmission. Swab and serum samples were transported on ice within 24 hours to the Washington Animal Disease Diagnostic Laboratory (WADDL) for PCR and antibody testing.

### Testing

We performed RNA extraction and SARS-CoV-2 real-time reverse transcription PCR (RT-PCR) for the SARS-CoV-2 RNA-dependent RNA polymerase gene (RdRp) as described for respiratory and fecal swab specimens (*20*). We also performed a second RT-PCR targeting the N1 region on the nucleocapsid gene as described for RdRp-detected samples (CDC 2019-Novel Coronavirus real-time RT-PCR [2019-nCoVEUA-01] (*21*). There was 100% concordance (agreement) between the RdRp PCR and N1 PCR. After initial viral detection by PCR, 3 dog samples and 1 cat sample were submitted to the University of Minnesota Genomics Center (Oakdale, MN, USA) for whole-genome sequencing (WGS) (*22*). A second cat sample was submitted to the USDA National Veterinary Services Laboratory (NVSL; Ames, IA, USA) for WGS. Mutational analysis was performed by using the GISAID EpiFlu Database CoVsurver: Mutation Analysis of hCoV-19 (*23*,*24*). We deposited all 5 sequences into GISAID (accession nos. EPI_ISL_7845315–8, and EPI_ISL_8897004) and assigned SARS-CoV-2 lineages by using the PANGO lineage tool (*25*,*26*).

### SARS-CoV-2 Spike Protein Receptor Binding Domain ELISA

WADDL developed canine and feline SARS-CoV-2 ELISAs by using recombinant SARS-CoV-2 spike receptor‒binding domain (S-RBD) protein as antigen. The recombinant S-RBD protein was obtained from the University of Washington Center for Emerging and Reemerging Infectious Disease Laboratory of Wesley Van Voorhis through an institutional material transfer agreement. WADDL used an in-house standard operating procedure for indirect ELISA of SARS-CoV-2 in 96-well format based on a previous report for humans (*27*).

The major components of the assay were recombinant S-RBD coating of plates as target antigen (2 μg/mL in carbonate-bicarbonate buffer; Sigma-Aldrich, https://www.sigmaaldrich.com); a 1:100 dilution of test serum diluted in ChonBlock ELISA Buffer (Chondrex Inc., https://www.chondrex.com); anti-dog IgG‒horseradish peroxidase conjugated as linker (goat anti-canine IgG; Southern BioTech, https://www.southernbiotech.com); and the 3,3′,5,5′-tetramethylbenzidine liquid substrate system (Sigma-Aldrich) to develop the optical density (OD). Plates were blocked with ChonBlock ELISA buffer per the manufacturer’s instructions, washing solution consisted of phosphate-buffered saline plus 0.1% Tween 20 (Sigma-Aldrich), and plates were read on a plate reader at 450 nM. Serum samples were tested in triplicate and used at the test OD.

For the dog RBD ELISA, negative controls consisted of serum samples collected from 6 dogs before COVID-19, archived at WADDL and tested for 5 canine viruses: adenovirus, distemper virus, coronavirus, parainfluenza, and parvovirus. All 6 samples had antibody on >1 of the tests performed. However, no serum sample reacted in the SARS-CoV-2 canine RBD ELISA.

For the cat RBD ELISA, negative controls consisted of serum samples collected from 3 cats before COVID-19 from WADDL archives and tested for feline coronavirus IgG and feline panleukopenia virus IgG. Two of the 3 samples had antibody on >1 of the tests performed (including 2 for feline coronavirus). However, neither sample reacted in the SARS-CoV-2 feline RBD ELISA.

We tested negative controls in triplicate and used the mean as the negative control OD. We used a ratio of test OD:negative control OD to determine the results. The positive cutoff of 2.0 test OD:negative control OD ratio equated to the mean of negative controls +3 SDs of the mean. Use of +2 or +3 SDs from the mean OD of defined negative control serum samples is a commonly used method when no standard negative or positive control serum samples are available. Use of + 3 SDs from the mean of defined negative control serum samples was chosen as the most conservative SARS-CoV-2 RBD ELISA cutoff to reduce the risk for false-positive results.

We performed SARS-CoV-2 RBD ELISA in triplicate on 3 different occasions for all samples and tabulated the final results as a mean value obtained from the repeated testing. Initially, because no dog or cat in Washington or Idaho had previously been confirmed to be SARS-CoV-2 seropositive, the first antibody-positive case for each species and state was sent to the USDA NVSL for confirmation by virus neutralization (VN) assay in keeping with regulatory recommendations. Subsequently a subset of 30 SARS-CoV-2 RBD ELISA‒positive serum samples that had a range of ELISA output (20 dogs and 10 cats) and 4 SARS-CoV-2 RBD ELISA‒negative serum samples (2 dogs and 2 cats) were compared by inter-laboratory comparison to live SARS-CoV-2 VN testing performed at the USDA NVSL. Although a VN test is not a validation of an ELISA because they detect different biologic functions of antibody that could involve different epitopes, avidity or affinity, the SARS-CoV-2 RBD ELISA to VN comparison showed 91% overall agreement (31/34), and a Cohen κ of 0.68 (substantial agreement), a metric that takes into account agreement by chance.

### Statistical Analyses

The primary aim was to estimate the burden of household SARS-CoV-2 transmission from humans to their pets. Secondary aims included describing the nature of human‒animal contact within households and identifying risk factors for household transmission, including human‒animal contact.

### Outcome

We defined animal infection or illness with SARS-CoV-2 as an animal meeting >1 of the following criteria: SARS CoV-2 RBD ELISA‒seropositive status, PCR-positive status, or illness consistent with SARS-CoV-2 infection, hereafter referred to as illness, defined as participant answer of yes to the survey question “Since the time of COVID diagnosis/symptom onset in the household, has this animal had any new issues with difficulty breathing, coughing or decreased interest in playing, walking, or eating?” We parameterized serostatus as ELISA ratio, log-transformed for interpretability, and PCR-positive status and illness as binary variables.

### Regression Models

We defined outcome as an animal case of SARS-CoV-2. Separate regression models were fit for each outcome definition.

Household-level exposures included residence in house versus apartment or condominium, home size in square feet, and the number of human confirmed SARS-CoV-2 cases. Animal-level exposures included sharing beds or bowls (separately) with human household members and SARS-CoV-2 positive household members taking precautions to prevent transmission to their pets. We also examined the association between canine seropositivity and illness compatible with SARS-CoV-2 infection in the animal and between seropositivity and time since the animal was first exposed, defined as 2 days before the first date any household member had symptoms of COVID-19 or a positive result.

**Figure 1:**
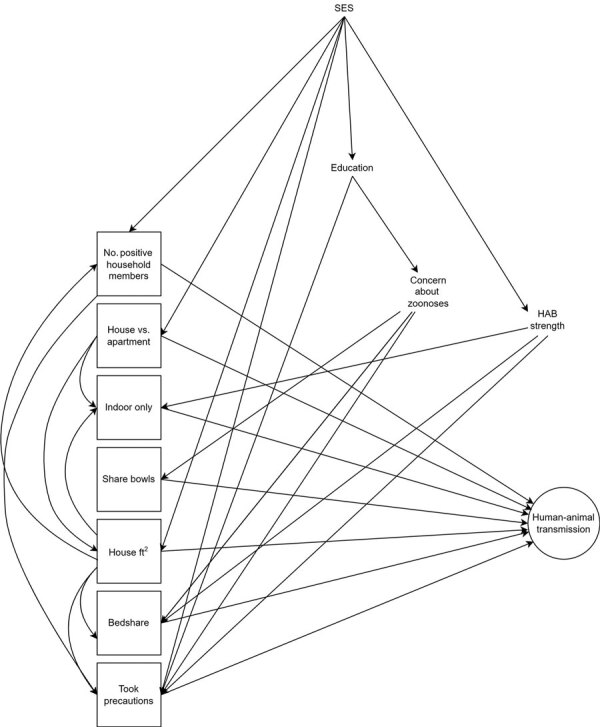
Directed acyclic graph for human‒animal transmission of SARS-CoV-2, Washington and Idaho, USA. Squares indicate exposures of interest and circles indicate outcomes (approximated by serostatus, PCR result, and illness in separate models). Measured and unmeasured confounders are included. SARS-CoV-2‒positive household member(s) took precautions to prevent transmission to pet. Indoor-only indicates the animal does not go outdoors; bedshare indicates the animal shares a bed with >1 household members. HAB, human‒animal bond; SES, socioeconomic status.

We identified possible confounders a priori by using a directed acyclic graph (Figure 1). We defined the minimum sufficient adjustment set by using this graph and appropriate software (DAGitty, http://www.dagitty.net) separately for each exposure (*28*). Animal species was explored as an effect modifier by using a multiplicative interaction term, and stratified results presented for all cases in which this interaction term reached statistical significance (p<0.05).

For each exposure of interest, we implemented a generalized estimating equation approach with an exchangeable working correlation structure, household as the clustering variable, and binomial models with a logit (binary outcomes) or Gaussian (continuous outcomes) link by using the geepack package in R (*29*). For regression of ELISA ratio on illness and time since first exposure, we performed linear regression by using the glm() function in R.

## Results

### Recruitment

**Figure 2:**
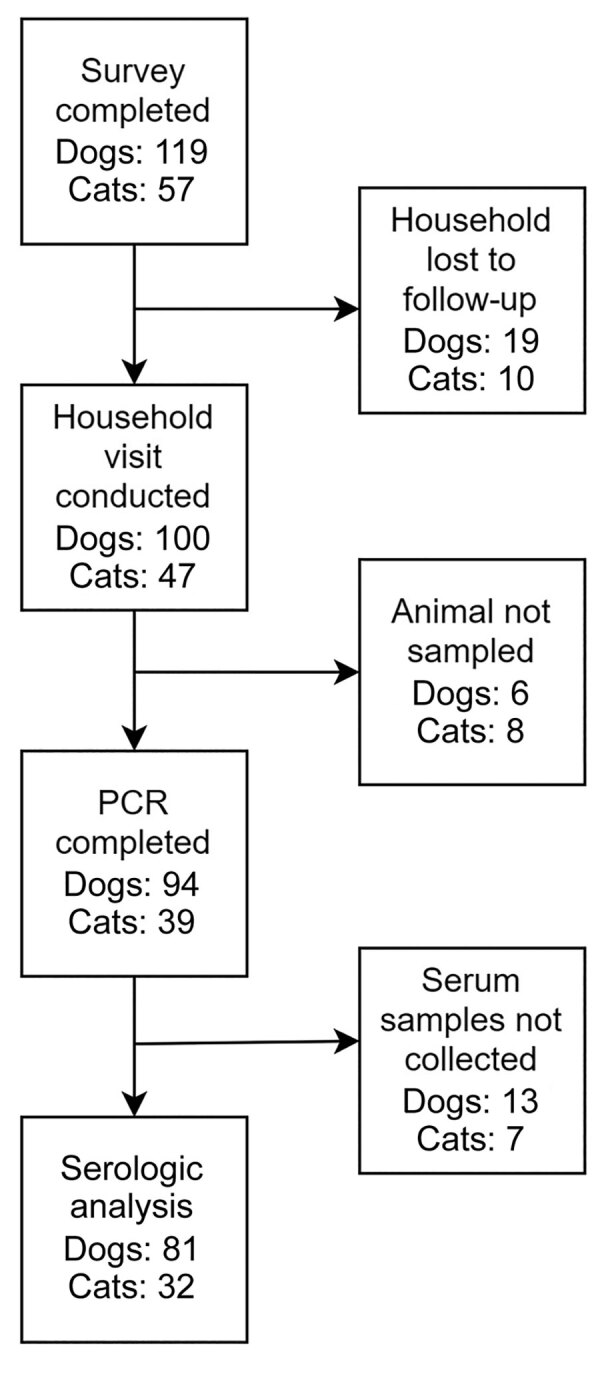
Flowchart indicating serologic and PCR sampling for study of household transmission of SARS-CoV-2 from humans to pets, Washington and Idaho, USA. Of 119 dogs and 57 cats corresponding to 105 households that had completed surveys, PCR testing was complete for 94 dogs and 39 cats, and serologic testing was complete for 81 dogs and 32 cats. The remaining pets were not sampled because of safety concerns.

A total of 107 eligible households enrolled and completed the survey; 83 households, corresponding to 100 dogs and 47 cats, had a sampling visit conducted (Figure 2). Of those animals, 6 dogs and 8 cats were not sampled because of temperament, leaving 94 dogs and 39 cats that had PCR results. An additional 13 dogs and 9 cats were safe to restrain for swab (PCR) samples but not for serum collection, leaving 81 dogs and 32 cats that had serologic results.

### Descriptive Statistics

**Figure 3:**
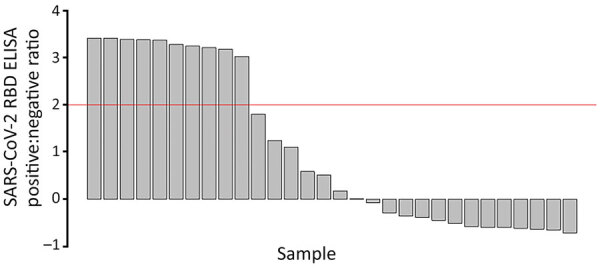
SARS-CoV-2 RBD ELISA serologic data for cats in study of household transmission of SARS-CoV-2 from humans to pets, Washington and Idaho, USA. PCR testing was complete for 39 cats, and serologic testing was complete for 32 cats. The remaining pets were not sampled because of safety concerns. Red line indicates cutoff value. RBD, receptor-binding domain.

**Figure 4:**
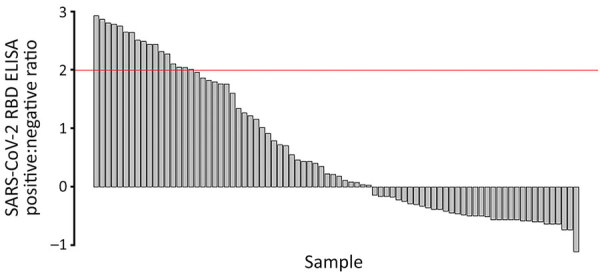
SARS-CoV-2 RBD ELISA serologic data for dogs in study of household transmission of SARS-CoV-2 from humans to pets, Washington and Idaho, USA. PCR testing was complete for 94 dogs, and serologic testing was complete for 81 dogs. The remaining pets were not sampled because of safety concerns. Red line indicates cutoff value. RBD, receptor-binding domain.

On average, at least 6 weeks (dogs) and 2 weeks (cats) elapsed between the last human COVID-19 diagnosis in the household and animal sampling (Table 1). Of the 119 dogs and 57 cats who had completed surveys, 20 dogs (20.4%, 95% CI 12.9%‒29.7%) and 19 cats (38.8%, 95% CI 25.2%‒53.8%) had reported illness. Of the 94 dogs and 39 cats who were PCR tested, 4 dogs (5.3%, 95% CI 1.8%‒12%) and 3 cats (7.7%, 95% CI 1.6%‒20.9%) were positive for any swab specimen; of the 81 dogs and 32 cats who had serum collected, 33 dogs (40.2%, 95% CI 29.6%‒51.7%) and 13 cats (40.6%, 95% CI 23.7%‒59.4%) were seropositive. SARS-CoV-2 RBD ELISA OD:negative control OD ratios in seropositive animals ranged from 2.03 to 21.22 for dogs (Figure 3) and from 3.01 to 30.35 for cats (Figure 4).

**Table 1:** Descriptive statistics for 119 dogs and 57 cats corresponding to 105 households for study of household transmission of SARS-CoV-2 from humans to pets, Washington and Idaho, USA*

Characteristic	Value	
	Dogs, n = 119	Cats, n = 57
Animal		
Illness consistent with SARS-CoV-2	20 (20)	19 (39)
Seropositive	33 (40)	13 (41)
PCR positive	5 (5)	3 (8)
ELISA ratio, mean (SD)	3.9 (4.93)	9.88 (12.51)
Activity during human quarantine†	33 (28)	7 (12)
Respondent took precautions‡	48 (41)	17 (30)
Age, y, mean (SD)	6.05 (3.86)	6.40 (4.50)
Male sex	66 (56)	28 (49)
Respondent aware of CDC guidelines§	62 (53)	29 (53)
Time from first diagnosis to sampling, d, mean (SD)¶	51.17 (60.64)	29.28 (19.17)
Time from last diagnosis to sampling, d, mean (SD)¶	43.06 (69.44)	15.16 (40.93)
Human		
Index case age, y, mean (SD)	41.78 (13.24)	47.91 (14.38)
Index case male sex	34 (29)	14 (25)
Index case underlying condition#	27 (23)	18 (32)
Index case was hospitalized	2 (2)	0
No. SARS-CoV-2‒positive household members, mean (SD)	1.78 (1.28)	1.72 (1.13)
No. household members who had COVID-19-like symptoms, mean (SD)**	0.27 (0.63)	0.26 (0.55)
No. household residents, mean (SD)	3.43 (1.49)	3.07 (1.28)
Environment		
Reside in a house	91 (76)	51 (89)
Reside in an apartment or condominium	51 (24)	6 (11)
Square footage of housing, mean (SD)	1,856.32 (932.74)	1,980.88 (1,095.15)
No. bedrooms, mean (SD)	3.24 (1.4)	3.19 (1.22)
No. of floors, mean (SD)	1.87 (0.82)	1.84 (0.62)
Access to outdoor space where pets can roam	99 (83)	41 (72)
Human‒animal contact		
Bowls used by animals cleaned in the kitchen	108 (91)	54 (95)
Humans and animals share bowls	15 (13)	8 (14)
Humans wash hands before handling animals	15 (13)	2 (4)
Humans wash hands after handling animals	50 (42)	12 (21)
Animal bedshares with humans	81 (69)	41 (73)
Animal shares a bedroom but not a bed with humans	54 (46)	19 (34)
Animal is indoor only	43 (37)	35 (61)
Animal sleeps outdoors	1 (1)	5 (9)
Humans pet the animal	117 (100)	56 (100)
Humans kiss the animal	88 (75)	38 (68)
Animal is allowed on furniture	101 (86)	56 (100)

*Values are no. (%) unless otherwise indicated. CDC, Centers for Disease Control and Prevention. †Activity is defined as going to a veterinary clinic or groomer; being walked off-leash; or visiting an off-leash park, dog park, kennel, or daycare facility. ‡Precautions to prevent human‒animal SARS-CoV-2 transmission following diagnosis: not petting or kissing the animal, staying in a different room, and having someone else feed and walk the animal. §Guidelines to prevent human‒animal SARS-CoV-2 transmission. ¶First diagnosis: earliest known, confirmed SARS-CoV-2 diagnosis in the household; final diagnosis: last known, confirmed SARS-CoV-2 diagnosis in he household. #Prexisting conditions: diabetes, kidney disease, heart disease, hypertension, immunosuppression. **Household members who had COVID-19-like symptoms but did not get tested.

Of the 94 dogs and 39 cats who were PCR tested, 5 dogs (cycle threshold [Ct] 26.0–37.67 for RdRp PCR and Ct 26.07–37.67 for N1 PCR) and 3 cats (Ct 27.03–39.97 for RdRp PCR and 27.03–39.97 for N1 PCR) were PCR positive by nasal/oropharyngeal swab specimens; 1 of these dogs was also PCR positive by a fecal swab specimen (Ct 39.20). Five PCR positive samples (2 cats and 3 dogs) had Ct values sufficient for WGS (Ct<30): The earliest cat sample (April 2021) that underwent WGS was in the Pango clade B.1.2. Another dog sample was identified as the Delta sublineage B.1.617.2.103 (AY.103), and the other 3 samples (1 cat and 2 dogs) were identified as Delta sublineage B.1.617.2.25 (AY.25). Of the 5 PCR-positive dogs, 3 were PCR positive before being seropositive and 2 were simultaneously PCR positive and seropositive.

There were 11 households that had >2 positive animals, and among multi-pet households that had >1 positive pet, mean prevalence (PCR or serology) was 91%. Of 8 PCR-positive cases, all were detected after April 2021, when the first case of the Delta variant was documented in Washington.

Nearly one third of dogs engaged in activities outside the household during periods of human isolation or quarantine. More than 50% of cats and dogs resided in households whose residents reported awareness of CDC guidelines to prevent human‒animal transmission of SARS-CoV-2, and 48 (41%) dogs and 17 (30%) cats resided in households that reported taking precautions to prevent transmission to household pet(s). No cats and only 2 dogs resided in a household in which an infected person was hospitalized for COVID-19. Nearly all dogs (83%) and most cats (72%) had access to yards or gardens and were allowed on furniture (86% of dogs and 100% of cats), and most dogs and cats were kissed by (75% of dogs and 68% of cats) and shared beds with (69% of dogs and 73% of cats) human household members. Almost all bowls for dogs (91%) and cats (95%) were washed in the kitchen.

### Regression Models

We calculated results of regression models as prevalence odds ratios for the binary outcome of illness, reflecting the cross-sectional design of this study, and as exp^β^ for the outcome of ELISA ratio, which can be interpreted as the relative change (ratio scale) in ELISA ratio for a 1 unit change in the exposure (Table 2). Because so few animals were PCR positive, we did not run regression models for that outcome. With the exception of house size, which was adjusted for house type because the minimum sufficient adjustment set was small for that exposure, confounders were not adjusted for because of concerns regarding overfitting arising from the small sample size. Effect modification by species was found only for house type.

**Table 2:** Regression model results for study of household transmission of SARS-CoV-2 from humans to pets, Washington and Idaho, USA*

Characteristic	Illness consistent with SARS-CoV-2, POR (95% CI)†	ELISA ratio, exp^β^ (95% CI)†
Exposure		
Indoor only	1.63 (0.77‒3.45)	1.07 (0.61‒1.88)
House type‡	0.52 (0.2‒1.34)	1.79 (1.02‒3.11) for dogs, 0.51 (0.25‒1.03) for cats
House square footage	1 (1‒1)	1 (1‒1)
Share bowls§	1.29 (0.39‒4.25)	1.78 (1.07‒4.49)
Bedsharing	1.48 (0.66‒3.33)	1.16 (0.68‒1.95)
Took precautions¶	0.71 (0.29‒1.75)	0.81 (0.48‒1.37)
No. SARS-CoV-2 infected humans	0.78 (0.54‒1.13)	1.18 (0.85‒1.64)
Illness consistent with SARS-CoV-2	Not examined	1.09 (0.59‒2.01)
Time since first exposure, days#	Not examined	1 (1‒1)

*House size was adjusted for house type, but no other models were adjusted for confounders due to overfitting concerns. POR, prevalence odds ratio. †Survey results were available for 119 dogs and 57 cats and serology results available for 81 dogs and 32 cats. ‡House versus apartment or condominium. §Animals and humans use the same bowls. ¶Precautions taken to prevent human-to-animal SARS-CoV-2 transmission after diagnosis: not petting or kissing the animal, staying in a different room, and having someone else feed and walk the animal. #First exposure was defined as 2 days before first positive diagnosis in the household or onset of symptoms, whichever was earlier.

Dogs residing in houses on average had a 79% (95% CI 2%‒211%) higher ELISA ratio than dogs residing in apartments or condos, and the inverse association was detected for cats (49% lower mean ELISA ratio, 95% CI 75% lower to 3% higher) and for the outcome of illness in both cats and dogs (48% lower prevalence odds, 95% CI 80% lower to 34% higher). This association reached statistical significance for dogs only. No other effect estimates reached statistical significance. However, there were positive trends across both outcome definitions for bed sharing with humans, sharing bowls, and being indoor only and a negative effect for precautions taken to prevent SARS-CoV-2 transmission after diagnosis. We also found that the ELISA ratio was positively associated with illness. However, we did not find evidence of an effect of time since first exposure on ELISA ratio or of house square footage on either outcome.

## Discussion

We present results of a cross-sectional, One Health study of SARS-CoV-2 transmission between persons and their pets. Results indicate that household transmission of SARS-CoV-2 from humans to animals occurs frequently, and infected animals commonly display signs of illness. We furthermore show that close human‒animal contact is common among persons and their pets in this study population, that this contact appears to enable SARS-CoV-2 transmission, and that pet owners are familiar with and willing to adopt measures to protect their pets from COVID-19. Virus-positive animal prevalence was >90% in multi-pet households that had >1 positive pet. Our results largely align with results from Canada (*30*) (positive effect for bedsharing in cats; 41% of dogs and 52% of cats seropositive; however, few PCR-positive pets) and studies from Texas (*31*) and Arizona (*32*) indicating that household pet interspecies transmission of SARS-CoV-2 is common.

The first limitation of our study is that several weeks had elapsed from first reported exposure to household sample collection from animals in most households, possibly limiting our ability to detect viral shedding by PCR testing but strengthening our ability to detect seroconversion. Second, although we assume transmission is from humans to pets, the cross-sectional nature of this study precludes certainty regarding the direction of transmission. Nevertheless, because SARS-CoV-2 is transmitted predominantly human-to-human, few cases of SARS-CoV-2 have been documented in dogs and cats, and no cases have been documented to be transmitted from dogs or cats to humans, we believe transmission in this study was exclusively from humans to pets. Third, our study is subject to residual confounding caused by inability to adjust for confounders without risking over-fitting. We do not expect unmeasured or unadjusted confounders to exert strong effects other than latent (and therefore difficult to measure and model) constructs, such as socioeconomic status, strength of the human–animal bond, and level of concern about zoonotic disease transmission. Fourth, our definition of illness in pets is simple and vulnerable to misclassification if these clinical signs are caused by other etiologies.

We believe respondents misunderstood the question “Is this animal indoor only vs. indoor/outdoor?” because 37% of dogs were reported to be indoor only. However, we believe that this variable retains its connection to degree of animal contact. We do not expect strong measurement error in any of the other variables examined. Because there is no standard for canine anti‒SARS-CoV-2 serology, validation of our ELISA was limited to analytic validation and we could not reliably estimate diagnostic sensitivity of our serologic test. Full diagnostic validation was not possible because of the absence of sufficient standard-positive and standard-negative samples, a limitation arising from emerging pathogen status of SARS-CoV-2. However, all pre‒COVID-19 samples evaluated were negative, indicating that specificity approaches 100%; all samples sent to the USDA NVSL for confirmatory PCR had concordant results; and a subset of 30 SARS-CoV-2 RBD ELISA‒positive serum samples that had a range of ELISA output; and 4 SARS-CoV-2 RBD ELISA‒negative serum samples showed substantial agreement with a virus neutralization test in an interlaboratory comparison with the USDA NVSL.

Although our primary aim, to estimate the burden of human-animal SARS-CoV-2 transmission, was estimated with reasonable precision, because of small sample size, variance was high for effect estimates produced by our regression model. By nature of our recruitment methods and study population, generalizability of our findings is probably limited to highly-educated, higher-income persons in urban and suburban communities.

In conclusion, our study contributes useful and novel findings to the literature on cross-species transmission of SARS-CoV-2, with relevance to other zoonoses transmitted in a household setting. In particular, our findings indicate households in this population are willing to adopt measures to protect their pets from SARS-CoV-2 infection and that these measures might be effective, indicating an opportunity to prevent household transmission of zoonoses through health education and policy.
